# Follistatin-Like 1 Is Downregulated in Morbidly and Super Obese Central-European Population

**DOI:** 10.1155/2018/4140815

**Published:** 2018-11-22

**Authors:** Martin Horak, Daniela Kuruczova, Filip Zlamal, Josef Tomandl, Julie Bienertova-Vasku

**Affiliations:** ^1^Department of Pathological Physiology, Faculty of Medicine, Masaryk University, Kamenice 5, Brno 625 00, Czech Republic; ^2^Research Centre for Toxic Compounds in the Environment, Faculty of Science, Masaryk University, Kamenice 5, Brno 625 00, Czech Republic; ^3^Department of Biochemistry, Faculty of Medicine, Masaryk University, Kamenice 5, Brno 625 00, Czech Republic

## Abstract

Follistatin-like 1 (FSTL1) is a secreted adipomyokine with a possible link to obesity; however, its connection to extreme obesity currently remains unknown. In order to analyze such association for the very first time, we employed a unique cohort of morbidly and super obese individuals with a mean BMI of 44.77 kg/m^2^ and measured the levels of circulating FSTL1. We explored the 3′ UTR of FSTL1 to locate a genetic variant which impairs microRNA binding. We located and investigated such SNP (rs1057231) in relation to the FSTL1 protein level, obesity status, and other body composition parameters. We observed a significant decline in FSTL1 level in obese subjects in comparison to nonobese ones. The evaluated SNP was found to correlate with FSTL1 only in nonobese subjects. The presented results were not affected by sex since both males and females expressed FSTL1 equally. We suggest that the FSTL1 decrease observed in extremely obese subjects is a result of adipogenesis reduction accompanied by a senescence of preadipocytes which otherwise willingly express FSTL1, increased adipocyte apoptosis, and epigenetic FSTL1 silencing.

## 1. Introduction

According to the latest WHO estimates for the European Union region, approximately 40–70% of adults are overweight and 10–30% are affected by obesity, i.e., an abnormal or excessive fat accumulation which constitutes a health risk. Obesity prevalence has tripled in many EU countries since the 1980s and this alarming number continues to rise. As a result, obesity is considered to be one of the greatest public health challenges of the 21st century, which is also evident from the fact that it consumes 4–7% of the total EU healthcare costs. It is responsible for increased morbidity and mortality and enhances the risk of developing metabolic and nonmetabolic disorders such as type 2 diabetes mellitus, glucose intolerance, or chronic low-grade inflammation and cardiovascular disease, cancer, glomerulopathy, or bone fragility, respectively.

It is a well-known fact that, far from being an inert fat deposit, adipose tissue constitutes an important and metabolically active endocrine organ which secretes proteins known as adipokines. To date, hundreds of adipokines have been identified and the vast majority of them, if not all, are linked to obesity [1]. Unfortunately, since current proteomic methodologies are not capable of describing pathophysiological pathways of obesity development on the level of individual adipokines, molecular approaches based on the study of a candidate molecule remain relevant. Although some adipokines, including, e.g., leptin, adiponectin, ghrelin, resistin, or interleukins have been studied extensively, the role of many others, including follistatin-like 1 (*FSTL1*), which has also been suggested as linked to obesity, remains unclear [2, 3].

*FSTL1* was first described in 1993 as *TSC-36* (TGFB-stimulated clone 36) in a study designed to identify genes regulated by transforming growth factor, beta [4]. It was later observed that *FSTL1* encodes a preadipocyte/adipocyte-secreted protein with a possible implication in obesity inflammation [5, 6]. Adipokine regulation is controlled by genetic as well as epigenetic factors such as microRNAs (miRNAs) which negatively regulate gene expression at the posttranscriptional level via miRNA–mRNA interaction [7]. MicroRNAs stand out as a contributing factor in obesity and other aspects of fat tissue biology by targeting genes involved in adipogenesis, chronic low-grade inflammation, or insulin resistance [8]. As mentioned above, a sufficient complementarity between miRNA and its targeted mRNA is required to achieve a proper regulation. This means that changes in DNA sequence and/or common sequence variants such as mutations and polymorphisms may result in the gain or loss of binding sites of specific miRNAs and thus result in novel phenotypic variations [9].

In view of the sizeable informational gap in the role of the FSTL1 protein in obesity, the main objectives of this study were to (1) analyze circulating FSTL1 protein levels in morbidly and super obese (OB) and nonobese (non-OB) individuals, (2) explore the 3′ UTR of the *FSTL1* gene using bioinformatic tools to locate genetic variants responsible for the gain or loss of specific miRNA binding sites, and (3) analyze the genotype frequencies of selected SNPs in the context of FSTL1 protein level, obesity status, and other anthropometric parameters in an OB and non-OB Central-European population.

## 2. Material and Methods

### 2.1. Study Subjects

A total of 133 unrelated Czech individuals of Caucasian origin were enrolled in this study. Written informed consent was provided by participants once they were fully informed of all study procedures. All experiments were conducted in adherence to the principles of the Declaration of Helsinki and all study protocols involving human subjects were approved by the Committee for the Ethics of Medical Experiments on Human Subjects of the Faculty of Medicine of Masaryk University in Brno, Czech Republic. All subjects were recruited for the study through a mass media campaign targeting the Central European population of the South Moravian Region of the Czech Republic, as described in our previous study [10]. Inclusion and exclusion criteria were adopted from a previous study [11].

The study group included 133 individuals (30 males and 103 females; mean age 47.12 years; age range 17.6–73.2 years). Analyzed characteristics included sex, age, height, weight, obesity status (BMI > 30 kg/m^2^), systolic and diastolic blood pressure, triceptal/biceptal/supraspinal/subscapular skinfold thickness, total body fat, total body water, hip circumference, waist circumference, hip-waist ratio, FSTL1 protein level, and SNP rs1057231 genotype. Body composition was assessed by bioelectrical impedance analysis using a single-frequency bioimpedance analyzer (Bodystat Ltd., Douglas, Isle of Man, UK) with the subject lying in a supine position. Height was measured using a calibrated stadiometer. Weight was measured using a precisely calibrated set of scales in light indoor clothes and without shoes.

### 2.2. Candidate Genetic Variants

We explored miRNA binding sites within the *FSTL1* gene for specific genetic variants using PartiGeneDB, an online database of miRNA-related SNPs [12]. Selection criteria for particular SNPs were based on (1) their location within abundantly expressed miRNA binding sites, (2) the ability of SNPs to cause the loss of a regular binding site or the gain of a new illegitimate binding site of specific miRNAs, (3) the SNP-related binding energy change of specific miRNAs, and (4) population frequency in the Caucasian population.

Based on our selection criteria, we selected an SNP (rs1057231) located in the 3′ UTR of the *FSTL1* gene. The G allele of this SNP results in the loss of a binding site for miR-1, miR-206, and miR-613, which was expected to result in the suppression of miRNA inhibition and subsequent increase in mRNA and protein levels.

### 2.3. PCR

DNA used in our study was extracted from 10 mL of subject saliva collected after 3 hours of fasting. Extraction was performed using a standard technique employing proteinase K. The investigated SNP was genotyped by a previously described technique using a standard PCR-based method with subsequent restriction fragment length polymorphism (RFLP) analysis [13]. Obtained restricted fragments were subsequently separated by electrophoresis on 3% agarose gels stained with ethidium bromide. Visualization was performed by UV illumination using an image analyzer (AlphaImager™ 1220; Alpha Innotech Corp., San Leandro, CA, USA). Genotyping reliability was assessed by double sampling in over 20% of all samples; no differences were observed. Moreover, to avoid possible false positives, quality control and negative controls were always used.

### 2.4. ELISA

Plasma levels of FSTL1 were measured in duplicate with a commercially available enzyme-linked immunosorbent assay kit (DuoSet, R&D Systems, MN, USA) in accordance with the manufacturer's instructions. When necessary, the samples were diluted 1 : 10 in a sample dilution buffer (5% BSA in PBS). Absorbance was measured on a Spectramax 340PC Microplate Reader (Molecular Devices, CA, USA).

### 2.5. Statistical Analysis

We first determined whether the genotype distribution is in the Hardy–Weinberg equilibrium using the *χ* ^2^ test and Fisher's exact test. Subjects were further categorized according to genotype (TT/TG/GG), sex (male/female), and obesity status (obese/nonobese), and independence between variables was tested using Pearson's *χ* ^2^ test and Fisher's exact test. Due to a negatively skewed distribution of FSTL1 protein levels, logarithmic transformation was employed, and data is therefore presented as geometric mean ± GSEM (geometric standard error of the mean) unless otherwise specified. Transformed data was notably closer to a normal distribution which facilitated the use of ANOVA (analysis of variance) for intergroup comparison. Data analysis was performed using R, v. 3.1.2 [14]. Values of *p* < 0.05 and 0.05 < *p* < 0.10 were considered statistically significant and borderline significant, respectively.

## 3. Results

### 3.1. Baseline Characteristics of Study Subjects

For baseline characterization of study subjects, reflecting demographic, anthropometric, and clinical parameters in relation to obesity status and sex, see Table 1. The asymptotic Pearson's *χ* ^2^ test and Fisher's exact test provided evidence that genetic distribution is in the Hardy–Weinberg equilibrium. The independence of variables was also confirmed by Pearson's *χ* ^2^ and Fisher's exact test.

### 3.2. FSTL1 Level Downregulation in Extreme Obesity

**Figure 1:**
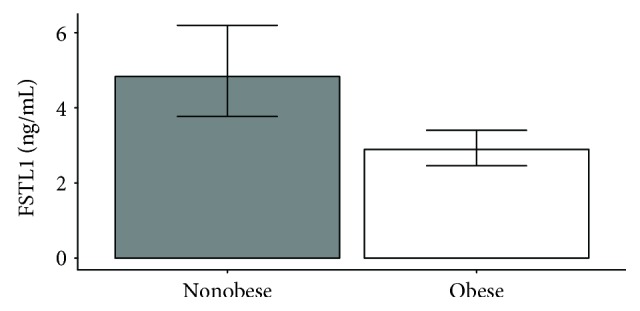
FSTL1 protein level in obese and nonobese participants. Quantification of FSTL1 protein levels obtained by ELISA kit for obese subjects (*n* = 81) and nonobese controls (*n* = 52), *p* = 0.044, Student's *t*-test.

We initially divided study participants into subcohorts and analyzed the FSTL1 protein levels according to obesity status, sex, genotype, and other body composition and anthropometric parameters listed in Table 1. We compared OB (BMI = 44.8 ± 6.0 kg/m^2^) and non-OB (BMI = 22.8 ± 2.3 kg/m^2^) individuals and observed a significant association, i.e., higher FSTL1 levels in the group of non-OB (FSTL1: OB vs non-OB, 2.89 ± 1.18 vs 4.83 ± 1.28, *p* = 0.044), see Figure 1. The FSTL1 level was 1.67 times higher for non-OB than for OB. Next, we compared males and females and observed no significant differences (FSTL1: males vs females, 3.63 ± 1.34 vs 3.51 ± 1.17, *p* = 0.924). Subsequently, we compared FSTL1 levels between males and females within OB (FSTL1 in OB: males vs females, 3.13 ± 1.44 vs 2.82 ± 1.20, *p* = 0.995) and non-OB (FSTL1 in non-OB: males vs females, 4.69 ± 1.63 vs 4.87 ± 1.34, *p* = 1.000) participants individually; however, we still observed no significant results. We also identified no significant differences in systolic and/or diastolic blood pressure, triceptal, biceptal, supraspinal, and subscapular skinfold thickness, total body fat, total body water, hip circumference, waist circumference, and hip-waist ratio between OB and non-OB individuals with respect to FSTL1 levels.

### 3.3. Association of the SNP with FSTL1 Protein Level

**Figure 2:**
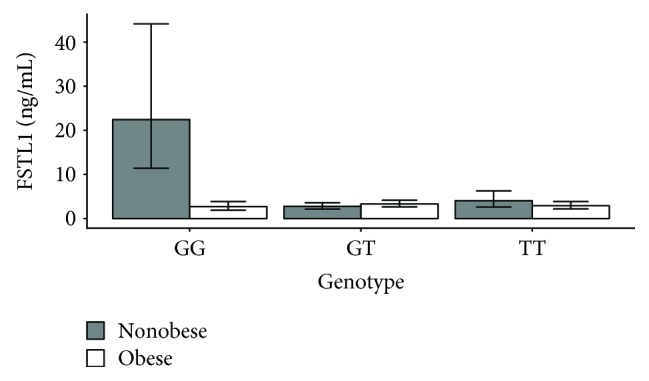
FSTL1 protein level association with genotype (rs1057231) in obese and nonobese participants. Quantification of the FSTL1 protein levels obtained by ELISA kit for obese subjects and nonobese controls with GG, GT, and TT genotypes. GG: *p* = 0.002; GT: *p* = 0.998; TT: *p* = 0.991, Student's *t*-test.

Next, we tested whether SNPs in the 3′ UTR of the *FSTL1* gene resulted in plasma level change as we expected based on bioinformatic predictions. Due to differences in expression between OB and non-OB individuals, we separated these two subcohorts and analyzed them separately. When assessing OB participants, we observed no significant association between FSTL1 levels and genotypes (FSTL1 in OB: GG vs GT vs TT, 2.69 ± 1.43 vs 3.30 ± 1.25 vs 2.89 ± 1.33). On the other hand, when we compared FSTL1 levels with genotypes in non-OB participants we observed a significant difference between GG and GT (FSTL1 in non-OB: GG vs GT, 22.41 ± 1.97 vs 2.76 ± 1.30, *p* = 0.003) and a borderline significant difference between GG and TT (FSTL1 in non-OB: GG vs TT, 22.41 ± 1.97 vs 4.03 ± 1.56, *p* = 0.083), see Figure 2. The FSTL1 level in non-OB individuals carrying the GG genotype was 8.12 times higher than in GT carriers and 5.56 times higher than in TT carriers.

### 3.4. Prediction Role of Obese Status and SNP to FSTL1 Protein Level

To identify genetic and nongenetic variables, we performed backward stepwise regression, i.e., a sequential procedure of removing one input variable at a time in order to build up a regression model in which the dependent variable (i.e., FSTL1 protein level) is characterized as the linear combination of independent variables (i.e., SNP genotype, obesity status, sex, and age). Using a multivariable regression model, we observed significant associations for obesity status and interaction of obesity status with genotype and no significant association for genotype (*p* = 0.227), sex (*p* = 0.924), and age (*p* = 0.924). We thus used a reduced model employing only genotype and obesity status as independent variables. Once we employed this model, we observed that both obesity status (*p* = 0.042) and the interaction of obesity status with genotype (*p* = 0.004) exert significant prediction roles for FSTL1 protein level.

## 4. Discussion

As worldwide obesity levels continue to increase, so does the need for understanding the underlying molecular mechanisms, including, e.g., the role of genes, genetic variants, and epigenetic factors involved in this condition. Although the FSTL1 protein has been described as an adipomyokine and although it has recently received a substantial amount of attention, only a limited number of studies have provided a possible link to obesity, namely by pointing out its role in inflammation and adipocyte cell fate determination and describing its methylation status in obese individuals [5, 15, 16]. In addition, respiratory quotient is the only obesity-related trait which was associated with a genetic variant within the FSTL1 gene so far by the means of GWAS [17]. As a result, the functional significance of FSTL1 remains elusive and the amount of currently available information on this topic remains limited. As FSTL1 has been described as functionally implicated in a range of processes including apoptosis, inflammation, or adipogenesis, it is not surprising that some of its functions may play a significant role in the obesity development [16, 18, 19]. However, since this role of FSTL1 has not been sufficiently addressed in existing publications, we decided to address the gap in order to provide information which could be helpful in both therapy and in interventions addressing obesity. We identified several associations of the FSTL1 protein level or its genetic variant with obesity.

First, to address the main point of this study, i.e., the FSTL1 difference between non-OB and OB participants, we used a control group and a unique cohort of morbidly and super obese. We observed a significant difference between these groups, with a higher plasma FSTL1 level in non-OB individuals. This result contradicts a study by Fan et al. who reported an increase of serum FSTL1 in 51 overweight/obese males [5]. To address the contradictory results, it is important to note that authors compared the control group with a mean BMI of 22.07 kg/m^2^ with a group of overweight/obese individuals with a mean BMI of 26.45 kg/m^2^. According to WHO, overweight corresponds to a BMI ranging from 25 to 30 kg/m^2^ and obesity to a BMI of over 30 kg/m^2^; a mean BMI of 26.45 kg/m^2^ thus suggests that only a small number of actually obese participants were present in the group of mildly overweight subjects. Therefore, the main strengths of our study include a bigger cohort with a mean BMI of 44.77 kg/m^2^, consisting of participants with type II and III obesity with BMI values of over 35 and 40 kg/m^2^ according to WHO, respectively, and cohorts composed of both females and males. We thus believe that our study provides more accurate results and thus a better reflection of molecular changes associated with obesity.

To date, several studies have identified diverse FSTL1 functions which may play a role in obesity and support our findings. First, it was discovered using both *in vitro* as well as *in vivo* approaches that FSTL1 is highly expressed in preadipocytes in comparison to adipocytes [20]. These results highlighted the possible role of FSTL1 in cell fate determination. While a subsequent study by Wu et al. confirmed its enrichment in mouse preadipocytes, the authors also observed drastic FSTL1 downregulation during the conversion of preadipocytes to adipocytes, where it became nearly undetectable [16]. The same result, i.e., a decrease of FSTL1 during and after adipogenesis, was reported again several years later [5]. Currently, most studies support the fact that obesity is accompanied by an overall adipogenesis reduction as well as by an increased number and mass of maturated adipocytes at the expense of preadipocytes [21]. Therefore, it is not surprising that we observed lower FSTL1 levels in morbidly and super obese individuals, who exhibit a decreased number of FSTL1 enriched preadipocytes. Another piece of the puzzle hinting at lower FSTL1 levels in obesity was provided by Oelsner et al. who observed a correlation between maternal obesity and increased FSTL1 methylation in children using saliva samples [15]. Very recently, FSTL1 gene methylation was also associated with its decreased expression [22]. However, no information is currently available on whether this methylation pattern remains stable as a child ages. In addition, while we have no information on the maternal obesity of our participants, it is well known that maternal obesity and family dietary patterns are one of the main factors of developing obesity in children and such patterns are often passed to descendants. Nevertheless, as increased FSTL1 methylation in obese individuals could further contribute to lower FSTL1 protein levels, it clearly deserves further attention. Next, several studies reported an antiapoptotic property of FSTL1 in many different cell types such as cardiomyocytes, dermal fibroblasts, neurons, endothelial cells, and lung and liver cancer cells [19, 23–27]. Moreover, it was revealed that its antiapoptotic molecular function works via the activation of AKT and inhibition of SMAD signaling [28, 29]. Both obese mice and humans have been reported to have increased adipocyte apoptosis accompanied by an upregulation of proapoptotic genes and marked downregulation of antiapoptotic genes [30]. Our results thus indicate that FSTL1 is probably only one of many downregulated antiapoptotic genes in obese individuals.

Last but not least, experiments on mice established the causative role of FSTL1 in inflammation and several other studies reported its upregulation in patients with inflammatory diseases [18, 31, 32]. Since obesity is characterized by a whole-body low-grade inflammation state, one may not be surprised by the previously reported upregulation of FSTL1 in overweight/obese individuals [5]. On the other hand, a study by Le Luduec et al. notes that FSTL1 was overexpressed in subjects with tolerated allogeneic heart transplant while the expression of inflammatory cytokines was attenuated [33]. Subsequent adenoviral FSTL1 overexpression *in vivo* produced the same results, i.e., prolonged allograft survival accompanied by proinflammatory cytokine inhibition, which suggests that FSTL1 regulation can be maintained in a context-dependent manner. Another possible reason for the FSTL1 decrease in morbidly and super obese is that obesity is associated with the increase of senescent cells in adipose tissue [34]. More specifically, it was reported that extremely obese individuals have over 30 times more senescent preadipocytes than nonobese individuals. Therefore, even though cellular senescence leads to a senescent phenotype with increased secretion of inflammatory cytokines, several authors reported that FSTL1 is significantly downregulated in senescent cells, where it is abundantly expressed under normal conditions, which can thus explain our observations [26, 34, 35].

According to our results, FSTL1 is not differentially regulated between both lean and obese males and females when assessed individually as it was reported in patients with osteoarthritis by Wang et al., who observed significantly higher FSTL1 levels in females in comparison to males [36]. On the other hand, controls in this study exhibited similar FSTL1 levels between sexes, which is consistent with our observation.

Finally, we analyzed the G/T polymorphism rs1057231 within the 3′ UTR of the *FSTL1* gene as—based on bioinformatic prediction—its G allele leads to the loss of a binding site for miR-1, miR-206, and miR-613. We confirmed our original hypothesis stipulating that the loss of a miRNA binding site results in protein level increase in the non-OB group. On the other hand, we did not observe a similar trend in OB individuals. We suggest two possible explanations for our observations. Firstly, FSTL1 downregulation in obese individuals is not achieved by miRNAs, which may work primarily as fine-tuners of gene expression. Therefore, this kind of SNP does not reflect an actual protein level in morbidly and super obese. Moreover, miRNAs were found to be deregulated in obesity, represented among others by downregulation of miR-206, which could further lower the impact of the evaluated SNP on final FSTL1 concentration [37]. One of the possible epigenetic regulation resulting in FSTL1 downregulation in obesity is methylation, as discussed above [15]. Secondly, an insufficient number of participants, especially males, for such genotype analysis might distort the final output. Therefore, our observations provide a preliminary data which require further independent replication samples and, therefore, we are preparing a validation study employing a larger cohort in order to convincingly confirm presented results.

In terms of future benefits for the population of obese patients, our results suggest that the FSTL1 protein may serve as a possible biomarker with predictive value with respect to future weight development of the patient. For instance, a decrease in FSTL1 levels could be used as one of the inclusion criterion for an earlier start of weight loss intervention as it can be expected that the weight loss will be more pronounced in the patients with lower baseline FSTL1 levels. Furthermore, the FSTL1 levels could be measured in the course of weight loss interventions to assess the degree of the patient's response and type and/or intensity of intervention can be adjusted accordingly. On the other hand, even though it was observed that the acute exercise temporarily increases the level of FSTL1, no data are currently available that would analyze the impact of prolonged exercise on FSTL1 levels [38]. Therefore, the longitudinal intervention studies are warranted. Last but not least, FSTL1 might also serve as a good marker of whole-body adiposity that does not necessarily have to correlate with BMI; however, further studies are needed to confirm this possibility.

## 5. Conclusions

In conclusion, it is biologically possible that overweight and mild obesity are associated with increased FSTL1 levels predominantly due to its proinflammatory action during this chronic low-grade inflammation state and a higher number of preadipocytes which willingly express FSTL1. On the other hand, morbid and super obesity are potentially associated with a decline in FSTL1 levels due to the continuous loss of adipogenesis and increased number of maturated adipocytes, increased cell senescence, enhanced demand for lowering FSTL1 antiapoptotic activity, and also possibly due to epigenetic gene silencing undertaken by methylation. In addition, these changes in FSTL1 protein levels are not affected by sex as both males and females in both the obese and nonobese groups expressed FSTL1 equally.

## Figures and Tables

**Table 1:** Study participant characteristics.

Parameter	Unit	Obese	Nonobese	Total	*p* value
		81	52	133	
Age	Years	52.5 ± 10.7	38.7 ± 10.8	47.1 ± 12.6	<0.001
Height	cm	166.4 ± 8.5	167.8 ± 8.9	166.9 ± 8.7	0.511
Weight	kg	124.1 ± 20.9	64.6 ± 10.8	100.8 ± 34.1	<0.001
BMI	kg/m^2^	44.8 ± 6.0	22.8 ± 2.3	36.1 ± 11.8	<0.001
Systolic blood pressure	mmHg	145.2 ± 19.5	116.4 ± 15.0	133.7 ± 22.7	<0.001
Diastolic blood pressure	mmHg	94.6 ± 13.0	77.3 ± 11.1	87.7 ± 14.9	<0.001
Triceptal skinfold thickness	mm	31.7 ± 6.4	18.0 ± 5.1	26.3 ± 9.0	<0.001
Biceptal skinfold thickness	mm	26.0 ± 8.0	11.3 ± 3.9	20.2 ± 9.8	<0.001
Supraspinal skinfold thickness	mm	32.1 ± 9.7	13.2 ± 4.2	24.3 ± 12.2	<0.001
Subscapular skinfold thickness	mm	35.4 ± 8.7	15.8 ± 4.8	27.6 ± 12.1	<0.001
Total body fat	%	49.1 ± 7.1	25.8 ± 7.2	40.0 ± 13.4	<0.001
Total body water	%	39.6 ± 5.1	54.2 ± 4.8	45.3 ± 8.7	<0.001
Hip circumference	cm	136.0 ± 12.4	97.6 ± 5.9	121.0 ± 21.4	<0.001
Waist circumference	cm	126.5 ± 13.5	77.8 ± 9.0	107.5 ± 26.7	<0.001
Hip-waist ratio	—	0.93 ± 0.10	0.80 ± 0.07	0.88 ± 0.11	<0.001
FSTL1	ng/mL	2.89 ± 1.18	4.83 ± 1.28	3.53 ± 1.15	0.044
FSTL1: genotype GG	ng/mL	2.69 ± 1.43	22.41 ± 1.97	5.24 ± 1.43	0.002
FSTL1: genotype GT	ng/mL	3.30 ± 1.25	2.76 ± 1.30	3.05 ± 1.18	0.998
FSTL1: genotype TT	ng/mL	2.89 ± 1.33	4.03 ± 1.56	3.24 ± 1.27	0.991

FSTL1 level is presented as a geometric mean ± geometric standard error of the mean; other parameters are presented as an arithmetic mean ± standard deviation.

## Data Availability

The data used to support the findings of this study are included within the supplementary information file.

## Abbreviations

FSTL1: Follistatin-like 1
miRNA: MicroRNA
OB: Obese
non-OB: Nonobese
SNP: Single-nucleotide polymorphism
UTR: Untranslated region.

## Supplementary Materials

Supplementary Materials Supplementary 1: contains raw data used for our analysis.
